# Predictive Correction Model for Corneal Back Surface Astigmatism With IOLMaster700 Keratometry Data in a Cataractous Population

**DOI:** 10.1111/ceo.70009

**Published:** 2025-10-25

**Authors:** Achim Langenbucher, Peter Hoffmann, Alan Cayless, Nóra Szentmáry, Kamran Riaz, Damien Gatinel, Oliver Findl, Seth Pantanelli, Tun Kuan Yeo, Giacomo Savini, Jascha Wendelstein

**Affiliations:** ^1^ Department of Experimental Ophthalmology Saarland University Homburg (Saar) Germany; ^2^ Augen‐und Laserklinik Castrop‐Rauxel Castrop‐Rauxel Germany; ^3^ School of Physical Sciences The Open University Milton Keynes UK; ^4^ Dean McGee Eye Institute University of Oklahoma Oklahoma City Oklahoma USA; ^5^ Rothschild Foundation Hospital Paris France; ^6^ Vienna Institute for Research in Ocular Surgery (VIROS) Hanusch Hospital Vienna Austria; ^7^ Department of Ophthalmology Penn State College of Medicine Hershey Pennsylvania USA; ^8^ Department of Ophthalmology Tan Tock Seng Hospital Singapore; ^9^ IRCSS Bietti Foundation Rome Italy; ^10^ Department of Ophthalmology Ludwig‐Maximilian‐University Clinics Munich Germany

**Keywords:** cross‐validation, keratometry, prediction of total corneal power, predictive correction model, total keratometry

## Abstract

**Background:**

To develop and validate various models to predict total keratometry (TK) power vector components TKC0 and TKC45 from classical keratometry (K) KC0 and KC45 based on a large dataset of pre cataract surgery IOLMaster 700 measurements.

**Methods:**

Retrospective cross‐sectional multicentric study evaluating a dataset containing 13 6378 IOLMaster 700 measurements including K and TK. Left eyes were mirrored about the facial axis. Based on 80% training data, we developed a global and segmented constant model (CM and CMS), a global and segmented (according to the angle A1 of the flat keratometric meridian) linear model (LM and LMS), a harmonic model (HM) and compared these to a classical constant (CMR) and linear models (LMR) segmented into with‐the‐rule, against‐the‐rule and oblique astigmatism. The performance was cross‐validated using the root‐mean‐squared model fit error (RMSE).

**Results:**

In the 20% test data, RMSE was 0.173 D before correction and was reduced by 40%–42% to 0.100 and 0.104 D with the correction models. The segmented models performed slightly better than the global models, and the linear models performed slightly better than the constant models. With the individually adjusted changepoints, the CMS and LMS performed slightly better than the reference models CMR and LMR. There was no systematic difference between the RMSE with training and test data, indicating no overfit of the models.

**Conclusion:**

As the performance is quite similar for all tested correction models, we recommend using a simple global constant model to predict TK vector components. This could easily be implemented in any consumer software.

## 1 Introduction

Classical standard keratometry measures the corneal front surface curvature to estimate total corneal power. Based on model assumptions represented in terms of a keratometric index, keratometry or corneal topography assumes a fixed ratio of front to back surface curvature in both cardinal meridians [1, 2, 3]. However, we know from Javal's rule that the corneal back surface does not show a fixed proportionality in all meridians. Since the radius of the back surface relative to the front surface is typically even steeper in the vertical meridian than in the horizontal meridian, the corneal back surface adds a variable quantity and quality of against‐the‐rule (ATR) astigmatism that is not represented in these standard keratometry measurements [4, 5, 6, 7, 8, 9, 10].

For clinicians, reliable data on total corneal astigmatism is essential, especially in the context of toric intraocular lens (IOL) implantation and refractive surgery. There are several options for anterior and posterior corneal power available to clinicians: (1) measure both the anterior and posterior corneal surfaces using modern Scheimpflug imaging [11, 12] or optical coherence tomography [13, 14], and analyze the equivalent and astigmatic power of each surface separately; (2) measure both surfaces and combine their data into a single total equivalent and astigmatic power; or (3) rely solely on traditional keratometric data and apply statistical corrections to account for the difference between keratometry and total corneal power. To assess correction strategies, corneal power has to be decomposed into power vector components. This means that instead of using the power in the flat and steep meridians together with their orientation angles, we transform corneal power into an equivalent power which represents the arithmetic mean of the power in both meridians and into the astigmatic power vector components C0 and C45 which represent the projections of the astigmatism onto the 0°/90° meridian and onto the 45°/135° meridian [1, 11, 13, 15, 16, 17, 18]. Recognising the 180° periodicity of astigmatism, the C0 component/C45 component is typically plotted on the *X*/*Y* axis in a double angle plot [18, 19].

Before analysing power vector data, it is helpful to consider symmetry conditions of corneal astigmatism with respect to the facial axis. For instance, the astigmatism of left eyes could be mirrored compared to right eyes. This could be expressed in a standard notation by Axis:=180‐Axis or in the power vector notation by flipping the sign of the C45 vector component C45:=‐C45 [15].

Since the first presentation of Javal's rule, as simplified by Grosvenor [4, 5, 6], several nomogram or predictive model corrections (e.g., Abulafia, Baylor, Goggin, Goggin scheme as modified by LaHood, or Homburg‐Adelaide nomogram) have been established in clinical routine, including (1) static vector corrections [1, 13, 18, 20]; (2) linear regression corrections defined either globally over the entire range of keratometric meridians (“global models”) or segmented/piecewise, in which the range is divided into a number of segments, with separate corrections applied to each (“segmented models”) e.g., the range could be divided into segments according to keratometric with‐the‐rule (WTR) astigmatism (flat meridian between 0° and 30° or between 150° and 180°), ATR (flat meridian between 60° and 120°), or oblique (OBL, flat meridian between 30° and 60° or between 120° and 150°) [7, 8, 21]; or (3) machine learning‐based corrections [16]. This arbitrary segmentation into WTR, ATR, and OBL astigmatism has been verified with clinical data [22, 23].

There are two complementary strategies for considering corrections for the corneal back surface astigmatism: (1) we could derive a correction that maps classical keratometry to total power [1, 2, 13, 16, 17, 18], and (2) we could develop a concept to map keratometric astigmatism to refractive cylinder, for example as derived from refractometry after implantation of a non‐toric intraocular lens in the eye [4, 8, 10, 24]. The first concept is restricted to the pure effect of the contribution of corneal back surface astigmatism, which is not described with keratometry [18], and the latter concept includes effects such as topographic changes due to cataract incision, or the effect of angle alpha, or the lens decentration or tilt on the refractive cylinder [4].

One purpose of the present study was to extract and analyse the power vector components of keratometry and total keratometry (TK) in a large dataset derived with the IOLMaster 700 in a cataractous population after considering mirror symmetry of corneal astigmatism (mirroring left eyes). Another was to develop several predictive correction models to predict the total corneal power from the keratometric power using global and segmented strategies of predictive model corrections based on constant models, linear regression models, and a harmonic model, including optimisation of the change points between segments. The final purpose of the study was to validate these models using strict cross‐validation and to compare with a classical constant and linear regression model segmented in terms of keratometric WTR, ATR, and OBL astigmatism.

## 2 Methods

### 2.1 Dataset for Our Evaluation

A large dataset containing *N* = 136 378 measurements taken prior to cataract surgery with the IOLMaster 700 (Carl‐Zeiss‐Meditec, Jena, Germany) on eyes scheduled for implantation of an IOL and without a history of previous eye surgery was considered in this study. This multicentre dataset contains biometric data including complete measurements of eye laterality, keratometry, and total keratometry from 8 clinical centres: (1) Rothschild Foundation, Paris, France (Prof. Gatinel, *N* = 48 335); (2) Augen‐und Laserklinik Castrop‐Rauxel, Castrop‐Rauxel, Germany (Dr. Hoffmann, *N* = 24 992); (3) Department of Ophthalmology, Ludwig‐Maximilian University, Munich, Germany (Prof. Priglinger, Dr. Wendelstein, *N* = 17 305); (4) Dean McGee Eye Institute DMEI, Oklahoma City, USA (Prof. Riaz, *N* = 17 041); (5) Hanusch‐Clinics, Vienna, Austria (Prof. Findl, *N* = 16 290); (6) Department of Ophthalmology, Penn State College of Medicine, Hershey, USA (Dr. Pantanelli, *N* = 5080); (7) Department of Ophthalmology, Tan Tock Seng Hospital, Singapore (Dr. Yeo, *N* = 4817); (8) IRCCS Bietti Foundation, Rome, Italy (Dr. Savini, *N* = 2518).

The local ethics committee (IRB) has provided a waiver for this study (Ärztekammer des Saarlandes, 157/21), as all data processed in this study were already anonymised at source before being transferred to us for processing. This precludes any back‐tracing of the identity, and therefore informed consent of the patients was not necessary. Data tables were reduced to the relevant parameters required for our data analysis, consisting of the following measurements: Clinical centre, sex (female or male), laterality (right or left eye), keratometry data for the corneal front surface consisting of radius in the flat meridian (R1 in mm), radius in the steep meridian (R2 in mm), and the orientation angle of the flat meridian (A1 in degrees), and finally total keratometry data for the corneal front surface consisting of radius in the flat meridian (TR1 in mm), radius in the steep meridian (TR2 in mm), and the orientation angle of the flat meridian (TA1 in degrees).

The measurement data were anonymised at source and exported as .CSV map files using the software module for batch data export. The data were transferred to Matlab (Matlab 2022b, MathWorks, Natick, USA) for further processing.

### 2.2 Data Pre‐Processing in Matlab

Keratometric data (R1, R2, A1) and total keratometry (TR1, TR2, TA1) were decomposed into power vector components (KEQ, KC0, KC45 and TKEQ, TKC0, TKC45, respectively) using the Javal keratometer index (nK = 1.3375). Since we expect mirror symmetry with respect to the facial axis (vertical axis), the C45 components for left eyes were flipped in sign to consider both left and right eyes as right eyes [15]. Since we used the orientation angle A1 of the flat keratometric meridian for segmented analysis, eyes with identical measurements for R1 and R2 (non‐astigmatic eyes where A1 is, by default, set to zero by the software of the IOLMaster without clinical meaning) were excluded from the dataset.

**FIGURE 1:**
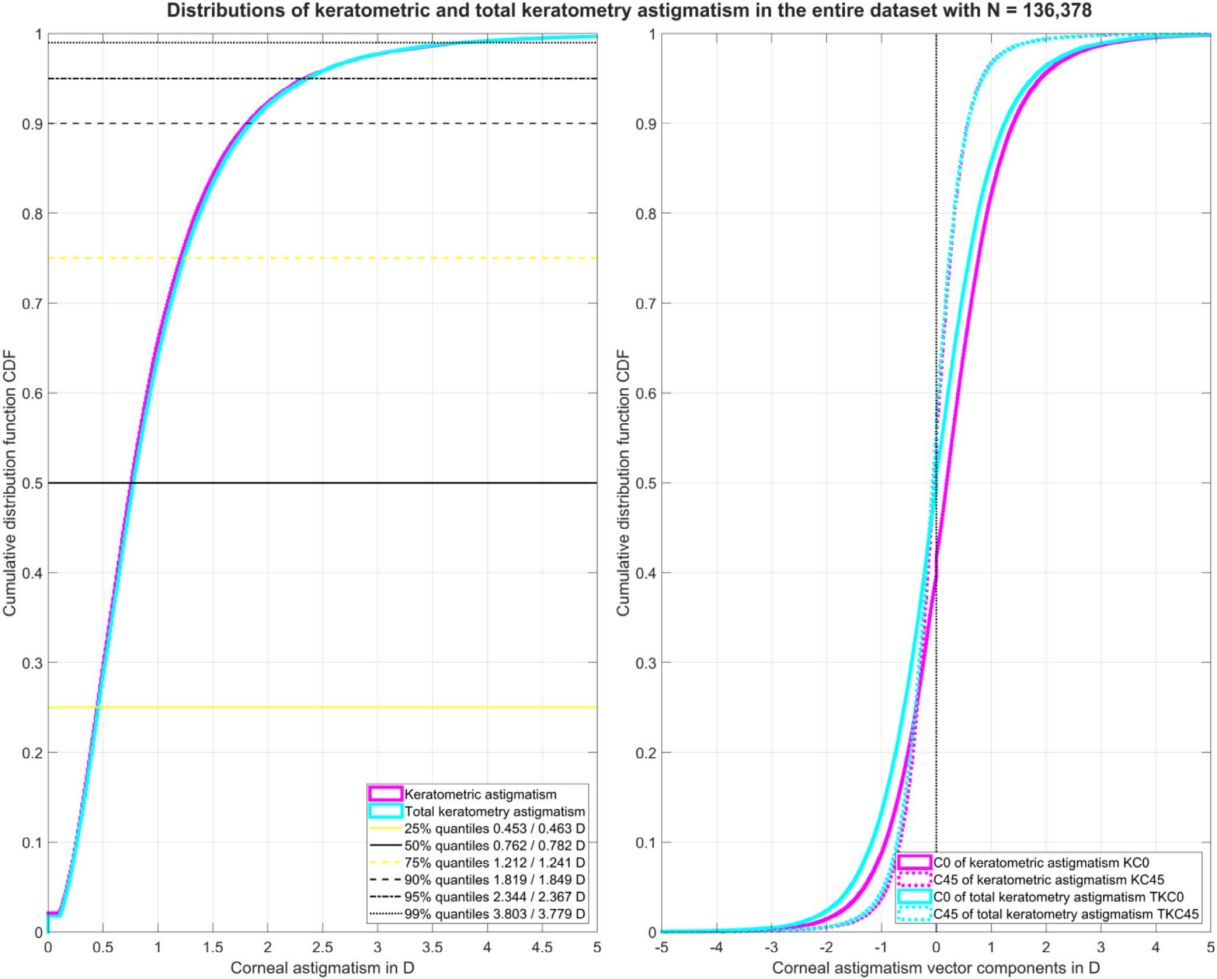
Cumulative distribution function (CDF) for the keratometric (K) and total keratometry (TK) astigmatism for the entire dataset before filtering out datapoints with a keratometric astigmatism of zero. The graph on the left shows the absolute value of the K and TK astigmatism, with the 25%, 50%, 75%, 90%, 95% and 99% quantiles listed in the legend. The graph on the right shows the CDF for the K and TK astigmatic power vector components C0 and C45. Please note that the KC0 is shifted slightly to the right (astigmatism with‐the‐rule) whereas KC45, TKC0 and TKC45 are mostly symmetric about zero (as marked with the black dotted line).

Figure 1 displays the cumulative distribution function for the keratometric and total keratometry astigmatism (left graph) together with the cumulative distribution function for the corresponding astigmatic power vector components KC0/KC45 and TKC0/TKC45 (right graph) for the entire population (*N* = 13 6378 measurements). The 25%, 50%, 75%, 90%, 95%, and 99% quantiles for the keratometric/total keratometry astigmatism are listed in the legend of the left graph. In 74.78%/74.32% of eyes, keratometric/total keratometry astigmatism was ≥ 0.5 D, in 36.39%/37.61% of eyes, it was ≥ 1.0 D, and in 8.21%/8.35% of eyes, it was ≥ 2.0 D.

The entire dataset was randomly split into training data (80%) and test data (20%), and the models described in the following section were developed using the training data, and their performance was then evaluated using the test data.

### 2.3 Models Describing the Difference of Total Keratometry and Keratometry

The simplest model that describes the astigmatic vector component differences between total keratometry (TKC0 and TKC45) and keratometry (KC0 and KC45) is a constant model (CM) fitted globally over the entire range of the flat keratometer axis A1. This model is defined as

$$\left[\begin{array}{c} \mathrm{TKC}0\\ \mathrm{TKC}45 \end{array}\right]=\left[\begin{array}{c} \mathrm{KC}0\\ \mathrm{KC}45 \end{array}\right]+\left[\begin{array}{c} \text{ICMC}0\\ \text{ICMC}45 \end{array}\right]+\delta =\left[\begin{array}{c} \mathrm{KC}0\\ \mathrm{KC}45 \end{array}\right]+\mathrm{ICM}+\delta$$

where ICM refers to the 2 component intercept vector of the correction, and δ to the fit error to be minimised.

In a more general case, a segmented constant model (CMS) was defined in which the astigmatic vector component differences between total keratometry and keratometry are piecewise constant for clusters of A1. If we assume M changepoints (CP) within the range of A1 (0° to 180°), together with the 180° periodicity of A1, we obtain M different segments, each with a separate constant model intercept ICMS1 to ICMSM. This model is defined as

$$\left[\begin{array}{c} \mathrm{TKC}0\\ \mathrm{TKC}45 \end{array}\right]=\left[\begin{array}{c} \mathrm{KC}0\\ \mathrm{KC}45 \end{array}\right]+\left\{\begin{array}{c} \begin{array}{c} \text{ICMS}1\\ \text{ICMS}1 \end{array}\\ \begin{array}{c} \ldots \\ \text{ICMSM} \end{array} \end{array}\right\}+\delta$$

In this model, in addition to the intercept vectors we also need to derive a suitable number of change points, M, and their locations, in order to minimise the fit error, *δ*.

Next, we defined a simple linear model (LM) in which the astigmatic vector component differences between total keratometry and keratometry are fitted globally over the entire range of A1. This model is characterised by an intercept (ILM) as well as a slope (SLM) as

$$\begin{array}{c} \left[\begin{array}{c} \mathrm{TKC}0\\ \mathrm{TKC}45 \end{array}\right]=\left[\begin{array}{c} \mathrm{KC}0\\ \mathrm{KC}45 \end{array}\right]+\left[\begin{array}{c} \text{ILMC}0\\ \text{ILMC}45 \end{array}\right]+\left[\begin{array}{cc} \mathrm{SLM}11 & \mathrm{SLM}12\\ \mathrm{SLM}21 & \mathrm{SLM}22 \end{array}\right]\cdot \left[\begin{array}{c} \mathrm{KC}0\\ \mathrm{KC}45 \end{array}\right]\\ \;+\delta =\left[\begin{array}{c} \mathrm{KC}0\\ \mathrm{KC}45 \end{array}\right]+\mathrm{ILM}+\mathrm{SLM}\cdot \left[\begin{array}{c} \mathrm{KC}0\\ \mathrm{KC}45 \end{array}\right]+\delta \end{array}$$

Again, in a more general case, a segmented linear model (LMS) was defined in which we assume that the astigmatic vector component differences between total keratometry and keratometry are piecewise linear for clusters of A1. If we define M changepoints within the range of A1, this results in M different segments, each with a separate linear model intercept (ILMS1 to ILMSM) and slope (SLMS1 to SLMSM). This model is defined as

$$\left[\begin{array}{c} \mathrm{TKC}0\\ \mathrm{TKC}45 \end{array}\right]=\left[\begin{array}{c} \mathrm{KC}0\\ \mathrm{KC}45 \end{array}\right]+\left\{\begin{array}{c} \begin{array}{c} \text{ILMS}1\\ \text{ILMS}1 \end{array}\\ \begin{array}{c} \ldots \\ \text{ILMSM} \end{array} \end{array}\right\}+\left\{\begin{array}{c} \begin{array}{c} \text{SLMS}1\\ \text{SLMS}1 \end{array}\\ \begin{array}{c} \ldots \\ \text{SLMSM} \end{array} \end{array}\right\}\cdot \left[\begin{array}{c} \mathrm{KC}0\\ \mathrm{KC}45 \end{array}\right]+\delta$$

In this model, in addition to the intercept vectors and slope matrices, we also have to derive a suitable number of changepoints, M, and their locations, in order to minimise the fit error *δ*.

Next, we defined a harmonic model (HM) in which the astigmatic vector component differences between total keratometry and keratometry are fitted globally with a sinusoidal modulation over A1. This model is characterised by an intercept (IHM) together with weights for the sine and cosine components (WHM) as

$$\begin{array}{c} \left[\begin{array}{c} \mathrm{TKC}0\\ \mathrm{TKC}45 \end{array}\right]=\left[\begin{array}{c} \mathrm{KC}0\\ \mathrm{KC}45 \end{array}\right]+\left[\begin{array}{c} \text{IHMC}0\\ \text{IHMC}45 \end{array}\right]+\left[\begin{array}{cc} \mathrm{WHM}11 & \mathrm{WHM}12\\ \mathrm{WHM}21 & \mathrm{WHM}22 \end{array}\right]\cdot \left[\begin{array}{c} \sin\left(A1\right)\\ \cos\left(A1\right) \end{array}\right]\\ \;+\delta =\left[\begin{array}{c} \mathrm{KC}0\\ \mathrm{KC}45 \end{array}\right]+\mathrm{IHM}+\mathrm{WHM}\cdot \left[\begin{array}{c} \sin\left(A1\right)\\ \cos\left(A1\right) \end{array}\right]+\delta \end{array}$$

The amplitudes for the C0 and C45 correction component are derived from $\text{AHMC}0=\sqrt{\mathrm{WHM}112+\mathrm{WHM}122}$ and $\text{AHMC}45=\sqrt{\mathrm{WHM}212+\mathrm{WHM}222}$, and the angles of the principal meridians are given by $\text{MHMC}0=\tan^{-1}\left(\frac{\mathrm{WHM}12}{\mathrm{WHM}11}\right)$ and $\text{MHMC}45=\tan^{-1}\left(\frac{\mathrm{WHM}22}{\mathrm{WHM}21}\right)$, respectively. With the amplitudes and angle data, the harmonic correction can be expressed as

$$\begin{array}{c} \left[\begin{array}{c} \mathrm{TKC}0\\ \mathrm{TKC}45 \end{array}\right]=\left[\begin{array}{c} \mathrm{KC}0\\ \mathrm{KC}45 \end{array}\right]+\left[\begin{array}{c} \text{IHMC}0\\ \text{IHMC}45 \end{array}\right]\\ \;+\left[\begin{array}{c} \text{AHMC}0\cdot \cos\left(A1-\text{MHMC}0\right)\\ \text{AHMC}45\cdot \cos\left(A1-\text{MHMC}45\right) \end{array}\right]+\delta \end{array}$$

As a reference, we defined a constant and a linear model based on the classical data clusters: a segmented constant model (CMR) was defined in which the astigmatic vector component differences between total keratometry and keratometry is piecewise constant for A1 clustered into wtr, atr and obl as

$$\left[\begin{array}{c} \mathrm{TKC}0\\ \mathrm{TKC}45 \end{array}\right]=\left[\begin{array}{c} \mathrm{KC}0\\ \mathrm{KC}45 \end{array}\right]+\left\{\begin{array}{c} \text{ICMRwtr}\\ \text{ICMRatr}\\ \text{ICMRobl} \end{array}\right\}+\delta$$

Finally, a segmented linear model (LMS) was defined in which we assume that the astigmatic vector component differences between total keratometry and keratometry are piecewise linear for clusters of A1 defined by wtr, atr and obl. For the 3 segments a separate linear model intercept (ILMRwtr, ILMRatr and ILMRobl, each are 2 × 1 vectors) and slope (SLMRwtr, SLMRatr and SLMRobl, each are 2 × 2 matrices). This model is defined as

$$\left[\begin{array}{c} \mathrm{TKC}0\\ \mathrm{TKC}45 \end{array}\right]=\left[\begin{array}{c} \mathrm{KC}0\\ \mathrm{KC}45 \end{array}\right]+\left\{\begin{array}{c} \text{ILMRwtr}\\ \text{ILMRatr}\\ \text{ILMRobl} \end{array}\right\}+\left\{\begin{array}{c} \text{SLMRwtr}\\ \text{SLMRatr}\\ \text{SLMRobl} \end{array}\right\}\cdot \left[\begin{array}{c} \mathrm{KC}0\\ \mathrm{KC}45 \end{array}\right]+\delta$$

All models were fitted en bloc using nonlinear iterative optimisation with the sequential linear programming (SQP) algorithm [25, 26]. The root‐mean‐squared (RMS) fit error (RMSE) derived from the vector length of the fit error *δ* $\left(\delta C02+\delta C452\right)$ was used as the optimisation metric.

## 3 Results

The dataset transferred to us originally contained *N* = 136 378 measurements. Of these, *N* = 133 502 (97.89%) having keratometric astigmatism > 0 were included in our data analysis. The dataset was randomly split into 80% training data (*N* = 106 802) and 20% test data (*N* = 26 700). The descriptive statistics for keratometry and total keratometry for the entire dataset, training dataset, and test dataset are listed in Table 1.

**TABLE 1:** Descriptive data for keratometry (K) and total keratometry (TK) after mirroring left eye data with respect to the facial axis for the entire dataset (upper block, after excluding the datapoints with keratometric astigmatism values of zero), and after randomly splitting into 80% training data (middle block) and 20% test data (lower block).

Data in dioptres		Keratometry K				Total keratometry TK			
		KEQ	K ast	KC0	KC45	TKEQ	TK ast	TKC0	TKC45
Entire dataset (*N* = 133 502)	Mean	43.0055	0.9694	0.2346	−0.0345	43.0564	0.9840	0.0402	−0.0618
	SD	1.6535	0.7794	1.0534	0.6174	1.6863	0.7825	1.0841	0.6325
	Median	43.0239	0.7757	0.2151	−0.0447	43.0851	0.7963	0.0064	−0.0682
	2.5% quantile	39.6749	0.1691	−1.7047	−1.1746	39.6397	0.1415	−1.9696	−1.2380
	97.5% quantile	46.1664	2.9564	2.4580	1.1596	35.2294	2.9373	2.3276	1.1605
Training data (*N* = 106 802)	Mean	43.0055	0.9594	0.2346	−0.0345	43.0564	0.9840	0.0402	−0.0618
	SD	1.6535	0.7794	0.0534	0.6174	1.6863	0.7825	1.0841	0.6325
	Median	43.0239	0.7757	0.2151	−0.0447	43.0851	0.7963	0.0064	−0.0682
	2.5% quantile	39.6749	0.1691	−1.7047	−1.1746	39.6397	0.1415	−1.9696	−1.2380
	97.5% quantile	46.1664	2.9564	2.4580	1.1596	46.2294	2.9373	2.3276	1.1605
Test data (*N* = 26 700)	Mean	43.0055	0.9694	0.2346	−0.0345	43.0564	0.9840	0.0402	−0.0618
	SD	1.6535	0.7794	1.0534	0.6174	1.6863	0.7825	1.0841	0.6325
	Median	43.0239	0.7757	0.2151	−0.0447	43.0851	0.7963	0.0064	−0.0682
	2.5% quantile	39.6749	0.1691	−1.7047	−1.1746	39.6397	0.1415	−1.9696	−1.2380
	97.5% quantile	46.1664	2.9564	2.4580	1.1596	46.2294	2.9373	2.3276	1.1605

*Note:* KEQ/K ast/KC0/KC45 refers to the equivalent power/astigmatism/astigmatic power vector component at the 0/90° meridian/astigmatic power vector component at the 45/135° meridian for keratometry, and TKEQ/TK ast/TKC0/TKC45 to the corresponding values for total keratometry. In the data table, we display the arithmetic mean, standard deviation (SD), median, and the lower (2.5% quantile) and upper (97.5% quantile) boundary of the 95% confidence interval.

**FIGURE 2:**
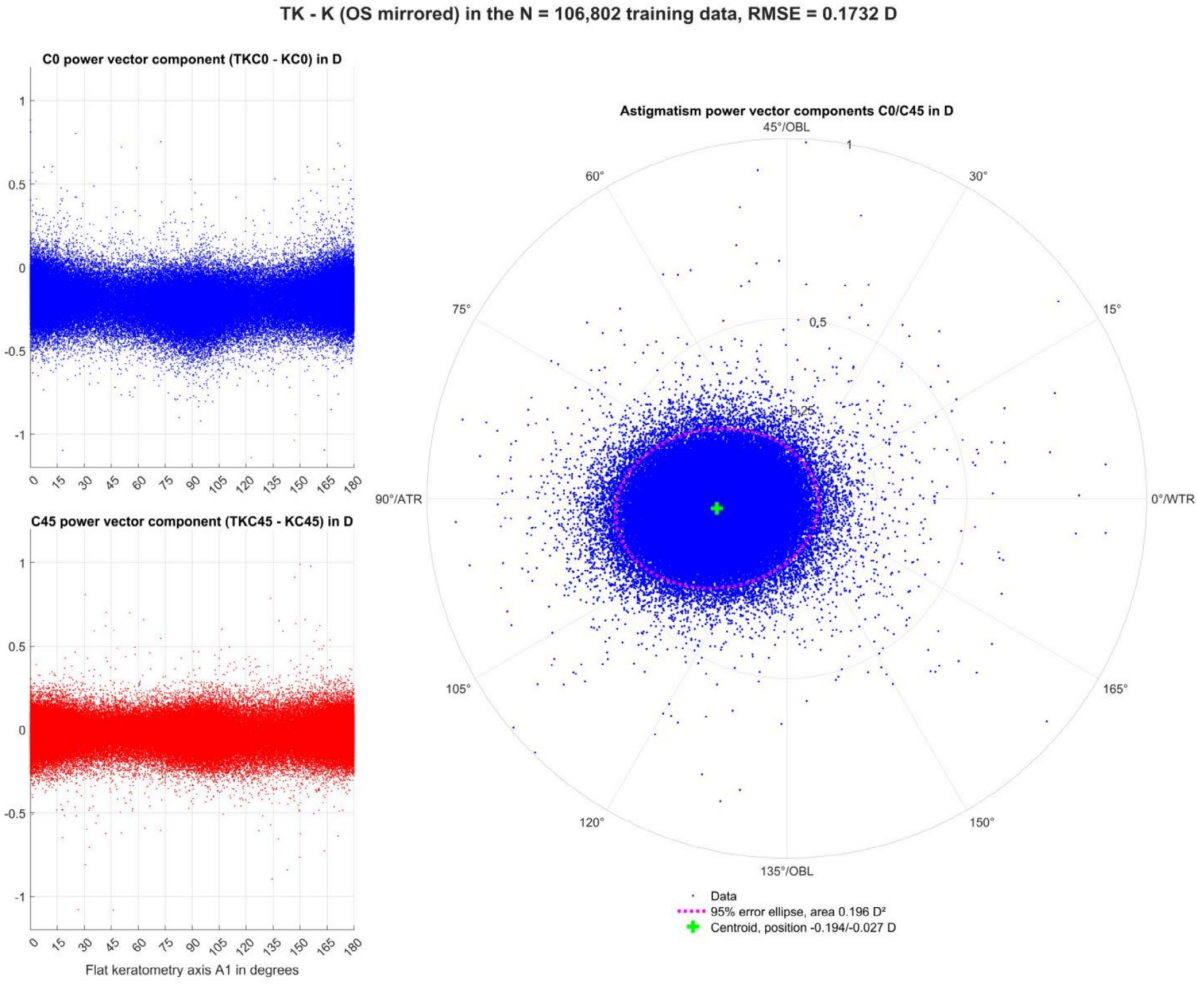
The scattergraphs on the left show the difference of total keratometry (TK) and keratometric (K) astigmatism power vector components (C0 in the upper graph and C45 in the lower graph) as a function of the K flat meridian angle A1 for the training data (with left eyes mirrored about the facial axis). The double angle plot in the right graph shows the angular distribution of both power vector components (TKC0‐KC0 and TKC45‐KC45) together with the centroid (location listed in the legend) and the 95% error ellipse (ellipse area listed in the legend) derived from the variance–covariance matrix. The shift of the C0 components towards negative values (in the upper left graph) corresponds to the shift of the distribution and centroid to the left (in the right graph).

The left‐hand side of Figure 2 displays scattergraphs for the training data showing the distributions of the astigmatic power vector components TK—K for C0 (upper graph) and C45 (lower graph) as a function of the angle A1 of the flat keratometric meridian. The graphs show that there is a concentration of datapoints around A1 = 0/180° and around A1 = 90°. The double angle plot for the difference TK—K is shown on the right, together with the centroid and the 95% error ellipse. This plot shows that there is a systematic offset towards negative C0 values and a slight offset towards negative C45 values. Due to mirror symmetry, we expect a slight shift towards positive C45 values in left eyes.

**FIGURE 3:**
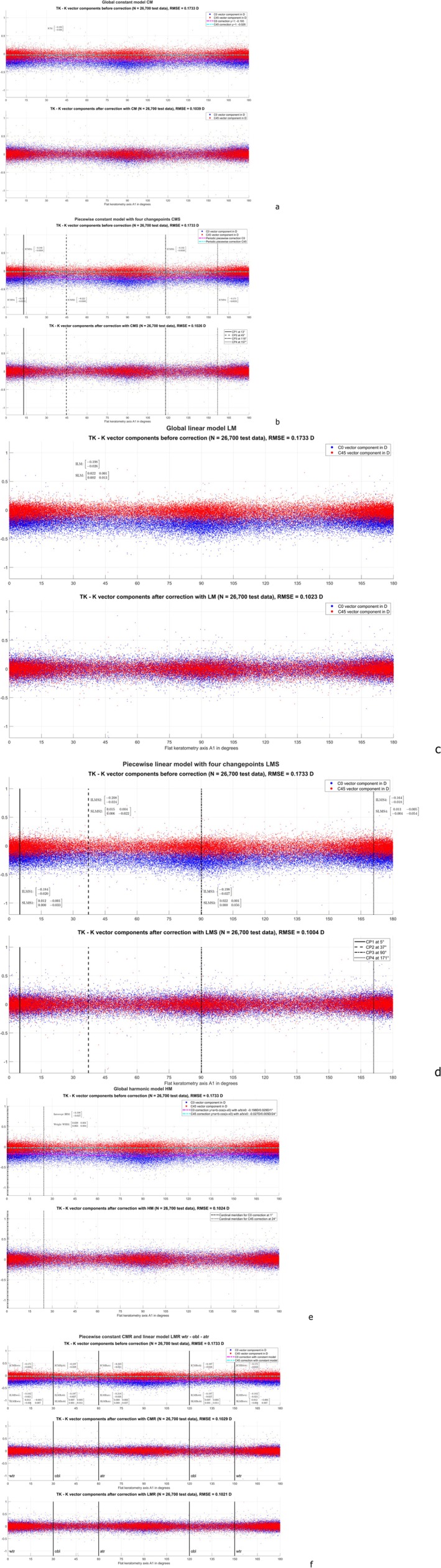
Scattergraphs for the test data (with left eyes mirrored at the facial axis) showing the difference of total keratometry (TK) and keratometric (K) astigmatism power vector components before (upper graph) and after correction (derived from the training data) (lower graph(s)) as a function of the flat keratometer meridian angle A1. (a) refers to the globally defined constant model CM, (b) to the piecewise (segmented) constant model (CMS), (c) to the globally defined linear model (LM), (d) to the piecewise linear model (LMS), (e) to the globally defined harmonic model (HM), and (f) to the constant (CMR, middle graph) and linear reference (LMR, lower graph) models, where the A1 segments were defined according to the classical standard into with‐the‐rule (wtr), against‐the‐rule (atr), and oblique (obl). The root‐mean‐squared model fit errors (RMSE) before and after correction are shown in the respective graphs. For the constant models (CM, CMS, CMR) the correction for the C0/C45 vector component is shown with dashdotted magenta/cyan lines. The intercept of the constant models, the intercept and slope of the linear models (LM, LMS and LMR), and the intercept and weight of HM are shown in the respective graphs.

The scattergraphs in Figure 3 show the differences in the C0 and C45 astigmatism power vector components of TK‐K before and after model corrections for the test data, the predictive models having been derived with the training data. Figure 3a shows the situation with the constant model CM, where the RMSE was reduced from 0.1733 D to 0.1039 D (in the training data from 0.1732 D to 0.1036 D). With this simple correction model, KC0/KC45 must be lowered by 0.193/0.026 D to map to TKC0/TKC45. Figure 3b shows the situation with the (piecewise defined) segmented constant model CMS. For consistency with the number of changepoints in a segmentation into wtr, atr, and obl, we decided to use 4 changepoints over the A1 range. As can be seen from the graph, the optimised changepoints are located at 13°/45°/118°/157° (instead of 30°/60°/120°/150° as used for wtr, atr, and obl). The RMSE was reduced to 0.1026 D (in the training data to 0.1023 D). The intercepts for the 4 segments (ICMS1 to ICMS4) are shown on the graph. Figure 3c shows the situation with the linear model LM, where the RMSE was reduced to 0.1023 D (in the training data to 0.1019 D). The intercept ILM and slope SLM are shown on the graph. Figure 3d shows the situation with the (piecewise defined) segmented linear model LMS. Again, for consistency with the number of changepoints in a segmentation into wtr, atr, and obl, we decided to use 4 changepoints over the A1 range. As can be seen from the graph, the optimised changepoints are located at 5°/37°/90°/171° (unlike the changepoints used for wtr, atr, and obl). The RMSE was reduced to 0.1004 D (in the training data to 0.1000 D). The intercepts (ILMS1 to ILMS4) and the slopes (SLMS1 to SLMS4) for the 4 segments are shown on the graph. Figure 3e shows the situation with the harmonic model HM, where a sinusoidal modulation of the correction over A1 is assumed. The graph shows that the model correction of the C0 component varies with an amplitude of 0.029 D around the intercept of −0.198 D with a maximal (negative) correction at 91°. The model correction for the C45 component does not show a systematic variation (amplitude of 0.005 D with a maximal (negative) correction at 114°) around the intercept of −0.027 D. The RMSE was reduced to 0.1024 D (in the training data to 0.1021 D). Finally, Figure 3f shows the performance of the constant and linear correction models with a classical data segmentation into wtr, atr, and obl. The intercept of CMR for the 3 relevant segments (ICMRwtr, ICMRatr, ICMRobl) and the intercept (ILMRwtr, ILMRatr, ILMRobl) and slope of the linear model LMR (SLMRwtr, SLMRatr, SLMRobl) are shown on the graph. With the CMR/LMR, the RMSE was reduced to 0.1029/0.1021 D (in the training data to 0.1025/0.1020 D).

## 4 Discussion

The main finding of our study was that, using the IOLMaster 700 in combination with the tested model corrections, the prediction error (RMSE) for total keratometry (TK) derived from standard keratometry could be systematically reduced from 0.17 D to approximately 0.10 D, depending on the correction model. This corresponds to a reduction of about 40% to 42%.

In general, the constant models CM, CMS, and CMR (RMSE reduction: 40.05%, 40.82%, and 40.61%, respectively) perform slightly worse than the linear models LM, LMS, and LMR (RMSE reduction: 41.00%, 42.09%, and 41.10%, respectively). The segmented models (CMS, LMS, and LMR) perform slightly better than the respective global models (CM and LM). Interestingly, the conventional segmentation into WTR, ATR, and oblique astigmatism, as used in several clinically established nomograms or predictive correction models [11, 18, 20, 22, 23, 27, 28, 29, 30, 31], performs slightly less accurately than models that allow individualised adjustment of segmentation changepoints.

Another interesting aspect is the mirroring of (in this study, left) eyes with respect to the facial axis [15]. If we assume mirror symmetry of keratometry or total keratometry, meaning that in normal cases the astigmatism of both eyes is similar in magnitude but with the axis reversed, that is, the (flat) axis of the left eye close to 180° minus the axis of the right eye, mirroring is mandatory to represent the situation of both eyes in a single prediction model [1, 2, 15, 18]. Alternatively, we could define separate models for left and right eyes [15, 18]. In the present study, we mirrored left eyes by flipping the sign of the C45 component for keratometry and total keratometry in order to treat all eyes as right eyes. We therefore ended up with a single model that could be used for both left and right eyes. In applying this model, it is important to remember that some corrections have to be made for left eyes: we could either (1) decompose keratometry into vector components, (2) flip the sign of C45, apply the correction, and flip the sign of C45 back, or (3) change the coefficients of the models when used for left eyes. For the intercept this means that the coefficient (1, 2) interacting with the C45 vector component has to be flipped in sign, and for the slope matrix of the linear models or the weight matrix for the harmonic model both off‐diagonal coefficients ((1, 2) and (1, 2)) have to be flipped in sign to be applied to left eyes.

Another notable aspect is that the conventional segmentation of the keratometric axis into WTR, ATR, and oblique includes two separate sectors for oblique axes—from 30° to 60° and from 120° to 150°. If mirror symmetry is not considered and both left and right eyes are included in the prediction model, there is a risk that any systematic tilt in the A1 vector (which exhibits mirror symmetry between left and right eyes) could cancel out across the dataset. Incorporating mirroring can help prevent this kind of neutralisation effect.

The results from the various models confirm that the C0 and C45 astigmatism power vector components cannot be treated separately. For instance, in the harmonic model, the cardinal meridians for the minimal and maximal corrections of the C0/C45 vector components are 91° and 1°/114° and 24°, respectively, and in the CMR or LMR models, we notice some crosstalk between the C0 and C45 astigmatism power vector components (off‐diagonal elements in the slope matrices).

These crosstalk effects between C0 and C45 are, however, quite low. For clinical routine, we require simple prediction models that can be easily implemented and provide clinical relevance. Given that the improvements in RMSE with all our models are quite similar, we feel that the simpler models would be preferred. For instance, the model with a constant vector correction is accurate, effective, and easy to implement in any consumer software or calculated with any simple pocket calculator. In the simplest version, to predict TK from K, we would subtract 0.198 from the C0 vector component and subtract (or add) 0.026 D to the C45 vector component for right (left) eyes. For easy implementation of the predicted total keratometry predTK (astigmatism predTKast and flat meridian angle predTKA1) from keratometry (astigmatism Kast with flat meridian angle A1) in any consumer software, the following symbolic code could be used as a template:

$$\begin{array}{ccc} \text{predTKast} & = & \text{Kast}2+\text{Kast}\cdot \left(\cos\left(\frac{\pi }{90}\right)\cdot ∆y+\sin\left(\frac{\pi }{90}\right)\cdot ∆x\right)+∆x\cdot ∆y\\ \text{predTKA}1 & = & \mathrm{mod}\left(\left(\frac{90}{\pi }\right)\cdot \text{atan}2\left(\frac{\text{Kast}\cdot \sin\left(\frac{\pi }{90}\right)+∆y}{\text{Kast}\cdot \cos\left(\frac{\pi }{90}\right)+∆x}\right)+180,180\right) \end{array}$$

where Δ*x* refers to the intercept of the C0 correction (−0.198), Δ*y* to the intercept of the C45 correction (−0.026 for right and +0.026 for left eyes), and cos/sin/atan to the cosine/sine/inverse tangent function. The atan2 function is available in most maths libraries and takes into account the sign of the numerator and denominator to avoid separate evaluations of the 4 quadrants. The modulo function (mod) shifts the axis range back to 0°…180°. Even the most advanced LMS model with individual definition of changepoints and linear correction where TK is predicted from an intercept ILMS and the K vector components weighted with slope coefficients SLMS individually adjusted in the 4 segments of A1 promises only a slight improvement.

Overall, comparing the model performance expressed in terms of RMSE fit error, we observe that the performance is quite similar when the model is applied to both the training and test data. This means that there is no overfitting in any of the prediction models based on our dataset.

However, our study has some limitations: first, this study relies on the assumption that the posterior cornea as measured by the IOLMaster 700 can be taken as a gold standard for the actual posterior corneal shape. However, this assumption may not be well supported by evidence in the literature. In fact, there is some discussion in the literature as to whether the posterior corneal shape predicted from keratometry may yield better results for toric IOL calculation than tomographic measurements in cases other than those following keratorefractive procedures. Second, we assumed mirror symmetry of the K and TK data with respect to the facial axis, which was fundamental to defining common prediction models for TK from K for both eyes. However, we feel that the benefit of a “universal” model for both eyes in terms of ease of use in the clinical setting justifies the use of these symmetry conditions. Third, our prediction models have been derived from a large dataset from the IOLMaster 700. Since we assume that keratometric and total power data from other devices, such as anterior segment tomographers or biometers, do not fully match the IOLMaster 700 data, our models are limited to predicting TK values specifically within the IOLMaster 700 context—for example, to support toric IOL planning when TK measurements are unavailable. Fourth, we restricted our analysis to a limited set of prediction models, including global and segmented constant and linear models, as well as a harmonic model. Other prediction models with a higher degree of complexity could be defined, but we should be aware that the risk of overfitting could increase systematically with the complexity of the model. And finally, the models developed in this study relate standard keratometry to total keratometry, but other factors such as lens tilt may be involved in predicting refractive astigmatism from standard anterior keratometry. These factors are, however, outside the scope of the current study.

In conclusion, we developed several predictive correction models to predict the total corneal power from the keratometric power based on a large dataset from the IOLMaster 700 derived before cataract surgery. Left eye data were mirrored with respect to the facial axis to derive a common correction for both eyes. We observed that all the models show quite similar performance and were able to reduce the RMSE by around 40% to 42%. The intuitive segmentation into with‐the‐rule, against‐the‐rule, and oblique astigmatism is a simplification, and it is possible that the performance could be marginally improved with an individual adjustment of the changepoints. Overall, as the implementation of (segmented) linear or harmonic correction models could be quite complex in clinical routine with only marginal improvements in performance, we recommend a correction with a simple globally defined constant model where a direct measurement of the corneal back surface curvature is unavailable.

## Conflicts of Interest

Dr. Langenbucher reports speaker fees from Bausch and Lomb and Johnson & Johnson Vision outside the submitted work. Dr. Hoffmann reports speaker fees from Hoya Surgical and Johnson & Johnson outside the submitted work. Dr. Szentmáry, Dr. Cayless, and Dr. Gatinel report no financial or proprietary interests. Dr. Riaz reports speaker fees from AbbVie, CorneaGen, and Bausch & Lomb; consulting fees from Ambrx Inc., Bausch & Lomb, Exelixis Inc., AbbVie, and Neumora Therapeutics; and research support from Nova Eye Medical. Oliver Findl is a scientific advisor to Carl Zeiss Meditec AG, Croma, and Johnson & Johnson. Dr. Pantanelli is a consultant for Bausch & Lomb and Carl Zeiss Meditec, and receives research support from Bausch & Lomb and Carl Zeiss Meditec, all unrelated to the present work. Dr. Tun Kuan Yeo reports research grants from Rayner, speaker fees from Alcon, Bausch and Lomb, Carl Zeiss Meditec, and Rayner outside of submitted work, and licences his intraocular lens formula to Bausch and Lomb, CSO, Intalight, Moptim, Topcon, and Towardpi. Dr. Savini reports speaker fees from Alcon, Moptim, SIFI, Thea, and Zeiss outside the submitted work. Dr. Wendelstein reports research grants from Carl Zeiss Meditec AG, speaker fees from Carl Zeiss Meditec AG, Alcon, Rayner, Bausch and Lomb, and Johnson & Johnson Vision outside of the submitted work. The authors declare no conflicts of interest.

## Data Availability

The data that support the findings of this study are available on request from the corresponding author. The data are not publicly available due to privacy or ethical restrictions.
